# Neuroimaging of Cerebral Small Vessel Disease and Age-Related Cognitive Changes

**DOI:** 10.3389/fnagi.2019.00145

**Published:** 2019-06-27

**Authors:** Michelle R. Caunca, Andres De Leon-Benedetti, Lawrence Latour, Richard Leigh, Clinton B. Wright

**Affiliations:** ^1^Division of Epidemiology and Population Health Sciences, Department of Public Health Sciences, Leonard M. Miller School of Medicine, Evelyn F. McKnight Brain Institute, University of Miami, Miami, FL, United States; ^2^Department of Neurology, Leonard M. Miller School of Medicine, University of Miami, Miami, FL, United States; ^3^National Institute of Neurological Diseases and Stroke (NINDS), National Institutes of Health, Bethesda, MD, United States

**Keywords:** aging, neuroimaging, cognitive aging, brain MRI, cerebrovascular disease

## Abstract

Subclinical cerebrovascular disease is frequently identified in neuroimaging studies and is thought to play a role in the pathogenesis of cognitive disorders. Identifying the etiologies of different types of lesions may help investigators differentiate between age-related and pathological cerebrovascular damage in cognitive aging. In this review article, we aim to describe the epidemiology and etiology of various brain magnetic resonance imaging (MRI) measures of vascular damage in cognitively normal, older adult populations. We focus here on population-based prospective cohort studies of cognitively unimpaired older adults, as well as discuss the heterogeneity of MRI findings and their relationships with cognition. This review article emphasizes the need for a better understanding of subclinical cerebrovascular disease in cognitively normal populations, in order to more effectively identify and prevent cognitive decline in our rapidly aging population.

## Introduction

Recent advancements in medicine, global health, and biotechnology have led to prolonged life expectancy, and thus, a rapidly aging population (Ortman et al., 2014). As a result, the prevalence of age-related diseases has increased, including cardiometabolic disease that impacts cerebrovascular health. Subclinical cerebral small vessel disease (CSVD) has garnered attention in recent years and is strongly related to modifiable risk factors such as hypertension and diabetes, especially in midlife (Debette et al., 2011; Power et al., 2017). Managing cardiometabolic factors has become a priority in maintaining optimal brain health (Gorelick et al., 2017). Many studies examining the association between CSVD and cognitive aging have used cognitive impairment or dementia as outcomes. We can improve our understanding of cognitive aging by distinguishing between changes in magnetic resonance imaging (MRI) that are associated with normal vs. abnormal cognitive performance. Studying those who have remained cognitively intact in old age can provide insight into the prevention of significant cognitive decline and dementia.

Neuroimaging features of CSVD include small subcortical infarcts and lacunes, white matter hyperintensities (WMH) on MRI (hypodensities on computed tomography), prominent perivascular spaces, cerebral microbleeds (CMBs), disruption of the blood-brain barrier (BBB), and diffusion-based markers of microstructural integrity (Alexander et al., 2007; Farrall and Wardlaw, 2009; Pantoni, 2010; Wardlaw et al., 2013b). These pathological entities form part of the American Heart Association (AHA)/American Stroke Association (ASA) definition of vascular contributions to cognitive impairment and dementia (VCID; Gorelick et al., 2011) and have been proposed as additions to recently published criteria for Alzheimer’s disease (Sweeney et al., 2019). Though several markers of CSVD have been related to an increased risk of cognitive impairment (Vermeer et al., 2007; Taheri et al., 2011a; Debette et al., 2019), their natural course across the lifespan and impact on age-related, non-pathological cognitive decline, remains to be elucidated. Furthermore, these CSVD markers have heterogeneous etiologies.

Data from neuroepidemiologic studies can help us understand and quantify the public health burden of CSVD and provide results that are more generalizable and reproducible than those from small, non-representative, or clinical samples (Paus, 2010; Falk et al., 2013; Ganguli et al., 2018). The characterization of CSVD markers from brain imaging in large epidemiologic studies has increased in recent years due to the availability of standardized, valid, and reliable post-processing software. This review article summarizes evidence from epidemiologic studies that investigate the relationship of these CSVD markers to cognition in older adults. In order to better characterize the role of CSVD in age-related cognitive changes, we restricted our review to studies of non-demented older adults when discussing cognitive performance or decline as the outcome. When describing the general mechanisms or epidemiology of the CSVD marker, we included studies with cognitively mixed samples, especially for CSVD markers for which there is a dearth of literature. Terminology of most CSVD markers discussed is derived from the STRIVE panel (Wardlaw et al., 2013b), which aimed to achieve a consensus on image interpretation, acquisition, and reporting on markers of CSVD based on neuroimaging and pathological characteristics. For each CSVD marker, we review the definition, etiology, and epidemiology, as well as summarize the evidence of its relation to cognitive function in non-demented samples. The goal of this review article is to inform future research in age-related cognitive decline, better differentiate between normal and pathological brain aging, and generate hypotheses for how CSVD may contribute to normal and pathological cognitive aging.

## White Matter Hyperintensities of Presumed Vascular Origin

### Definition and Measurement

Historically, white matter lesions (WML) were first observed on CT and were named “leukoaraiosis” (Hachinski et al., 1987). Once the use of MRI became widespread, these lesions were better known as WMH, due to their hyperintense appearance on T2-weighted sequences (Wardlaw et al., 2013b). The addendum “of presumed vascular origin” is used to distinguish them from white matter (WM) abnormalities secondary to other causes, such as multiple sclerosis or the leukodystrophies (Wardlaw et al., 2013b).

In the research setting, lesion severity is generally characterized either semi-quantitatively by visual grading or quantitatively by volume (e.g., WMH volume or WMHV). Volumetric quantification of WMHs is more reliable and sensitive than visual rating scales of WM lesions (van den Heuvel et al., 2006b) and has become more common in recent years due to the increased availability of various automated and semi-automated software. These systems quantify WMHV based on the pixel intensity of a determined area on T2-weighted images in either an automated or semi-automated fashion (DeCarli et al., 1999; Jenkinson et al., 2012). The transverse component of the MR magnetization decays as a result of spin-spin interactions and is characterized by relaxation of the time-constant, T2. A number of pathological processes, including edema, gliosis, and demyelination, leads to an increase in T2 and a relative increase of the signal intensity in a T2-weighted image; the pathology appears “hyperintense” when compared to homologous healthy tissue. Because cerebral spinal fluid (CSF) also appears bright on a T2-weighted image, confounding the identification of lesions (such as at the boundaries of the ventricles), fluid attenuated inversion recovery (FLAIR) prepared imaging is often used to null the signal from CSF, leaving predominantly parenchyma.

Often, lesions are further divided into periventricular WMH (PWMH), located adjacent to the lateral ventricular wall, or deep WMH (DWMH), located in the immediate subcortical region (Fazekas et al., 1987). Some studies have shown high correlations between the two types suggesting this division is arbitrary (DeCarli et al., 2005). However, other studies have found differences in pathological correlates when comparing PWMH with DWMH (Shim et al., 2015), supporting the use of this categorization. In statistical analyses, WMHV is usually modeled as a proportion of total intracranial volume to account for individual differences in head size. Alternatively, intracranial volume can be treated as an additional covariate in multivariable analyses. To model WMH burden, categories of WMHV (e.g., quartiles) are often used to classify participants with extensive or large WMH burden, but to date these categories have not been translated into clinically meaningful amounts and radiologists generally read scans qualitatively when examining these changes.

### Etiology and Pathological Correlates

WML are often ischemic in origin, developing as a consequence of arteriolosclerosis of the small arteries and arterioles that supply the WM, and damage to capillary beds and venous collagenosis has also been found (Moody et al., 1997; Pantoni, 2010; Wardlaw et al., 2015). Chronic ischemia results in vascular endothelial activation leading to the release of hypoxia-inducible factors, metalloproteinases (MMPs), and immunological mediators that cause hyaline wall thickening, smooth muscle cell loss, and narrowing of the vessel lumen (Fernando et al., 2006). These changes interfere with the auto-regulatory adaptations that would normally occur in response to attenuation of cerebral blood flow. Thus, focal infarction (lacunes) or areas of demyelination result and are then observed as WMH on MRI. Pathological studies suggest that WMH are comprised of discontinuous ependyma and gliosis of WM fibers with demyelination and axonal damage (Fazekas et al., 1993).

There are several contributors to the age-related cerebral ischemia that underlie WMH. Vascular pathologies, such as venous collagenosis and arteriolar tortuosity due to hypertension, develop with age and may reduce blood flow to downstream small vessels, inducing a hypoxic environment (Brown et al., 2002). More directly, hypertension has been robustly associated with greater WML load (van Dijk et al., 2008; Gottesman et al., 2010; Debette et al., 2011; Godin et al., 2011; Hajjar et al., 2011; Marcus et al., 2011; Verhaaren et al., 2013), presumably through the induction of small vessel hypertensive vasculopathy and subsequent vascular remodeling (Laurent and Boutouyrie, 2015), especially in the periventricular region. Hypertension may also cause vascular injury that results in BBB dysfunction (see below), increasing vascular permeability and causing cerebral edema and activation of astrocytes in the WM (Wardlaw et al., 2003). Relevant to cognitive aging, Alzheimer’s disease-related risk factors may also contribute to cerebral hypoperfusion. For example, epidemiologic evidence suggests that ApoE ε4 allele carriership and low serum amyloid levels are related to greater WML load (de Leeuw et al., 2004; Kaffashian et al., 2014). Low serum amyloid levels may reflect amyloid deposition in the cerebral vessel walls characteristic of cerebral amyloid angiopathy (CAA), which in turn reduces lumen diameter and diminishes blood flow (Bouras et al., 2006). Chronic ischemia alters mechanisms of perivascular lymphatic drainage of interstitial fluid, leading to the accumulation of amyloid and further exacerbating the hypoxic environment (Huang et al., 2010). More recent evidence from a smaller cognitively mixed study suggests that greater CSF Aβ_1–42_ is associated with lower WMH burden (Al-Janabi et al., 2018). Similar trends have been observed in amyloid PET imaging studies (Goodheart et al., 2015), though this may differ by APOE ε4 allele status (Noh et al., 2014). In another smaller study of β-amyloid positive individuals (as detected by PET imaging), APOE ε2 allele carriers exhibited greater WM lesion load compared to APOE ε4 allele carriers (Groot et al., 2018). The exact relationship between neurodegenerative and cerebrovascular pathology is still unknown, but most recent studies among predemented individuals suggest that CSF Aβ_1–42_ and WMH are associated with brain atrophy in an additive fashion (Bos et al., 2017).

In people with WMH, both BBB permeability and mean diffusivity are increased more than what would be expected by aging alone, even in areas of normal-appearing WM. The term WMH “penumbra” has been suggested to describe this, referring to decreased fractional anisotropy (FA) and higher mean diffusivity (MD) in apparently healthy tissues neighboring WMH (Maillard et al., 2011). Both FA and MD have been described as strong predictors of future stroke in healthy populations (Evans et al., 2016). This supports the notion that WMH are the most visible marker of a more diffuse process.

### Epidemiology

The association between WMHs of presumed vascular origin and greater age has been well-documented (Brickman et al., 2008; Ikram et al., 2008; Morris et al., 2009; Chowdhury et al., 2011). Other demographic factors have also been examined with age adjusted in multivariable models, suggesting that factors such as race/ethnicity and sex explain variability in WMHs above and beyond age. For example, WMH volume or grade was reported to be greater in racial/ethnic minorities when compared with non-Hispanic Whites in diverse population-based studies (Brickman et al., 2008; Knopman et al., 2011). In contrast, the multi-ethnic Omni cohort of the Framingham Heart Study demonstrated decreased WMH volume compared with the mostly Caucasian FHS Offspring cohort, but this may be due to the younger mean age of the Omni cohort (Stavitsky et al., 2010). Some studies also found greater WM disease in women compared to men (Yue et al., 1997; de Leeuw et al., 2001; van Dijk et al., 2008), though the exact mechanism of this sex difference is unknown.

### Cognition

Greater WML load has been related to worse general and domain-specific cognitive performance in community-based samples and several epidemiologic studies have specifically examined participants without cognitive impairment. Cross-sectional studies using data from non-demented participants and after adjusting for age have shown that greater WML load is associated with worse cognition (Koga et al., 2002; Au et al., 2006; Zhou et al., 2008; Godin et al., 2010; Vemuri et al., 2015), especially executive function (Lampe et al., 2019), processing speed (Au et al., 2006; van den Heuvel et al., 2006a; van Dijk et al., 2008; Raji et al., 2012; Knopman et al., 2015) and, to a lesser extent, episodic memory (Au et al., 2006). The associations with frontal lobe processes reflect damaged WM tracts relaying information to other parts of the brain.

Longitudinal analyses in non-demented people have also shown that those with greater WML load have significantly faster cognitive decline, similar in strength to the association of amyloid load to cognitive decline (Vemuri et al., 2015). Other longitudinal analyses have exhibited similar patterns, such that greater WML load is associated with greater cognitive decline, even after adjustment for age (Arvanitakis et al., 2016; Boyle et al., 2016).

Similar findings have also been demonstrated in cognitively normal participants who exhibit mild Parkinsonian signs (Camarda et al., 2018) and functional impairments (Murray et al., 2010; Wakefield et al., 2010; Dhamoon et al., 2018; Willey et al., 2018), further emphasizing the need to examine cognitively normal individuals who have other neurological deficits. Finally, region-specific WML load has also been examined in cognitively normal samples, and in particular, frontal lobe (Lampe et al., 2019) and periventricular (van den Heuvel et al., 2006a) WMLs are associated with worse cognitive performance. Generally, these associations are examined in multivariable models adjusted for age, suggesting that these associations are independent of aging.

Though these studies illustrate direct effects of WML load on cognitive performance, there are reports of indirect effects of WML load on cognition through markers of gray matter volume (Knopman et al., 2015; Rizvi et al., 2018). These data imply that cerebrovascular damage may cause degeneration of the surrounding brain parenchyma, potentially leading to cognitive impairment. However, the relationships between CSVD and neurodegeneration are complex and no consensus exists on whether the association is additive or synergistic.

## Lacunes of Presumed Vascular Origin

### Definition and Measurement

Lacunar infarcts are small subcortical infarcts that arise from occlusion of a single perforating cerebral artery (Fisher, 1982). Lacunar infarcts of presumed vascular origin have been further defined as round or ovoid cavities that are subcortical, fluid-filled, and measuring between 3 mm and about 15 mm in diameter (Wardlaw et al., 2013b). A peripheral hyperintense rim can often be observed on FLAIR sequences, although it is nonspecific and can also surround perivascular spaces (Wardlaw et al., 2013b). The distinction between lacunar infarcts and perivascular spaces is important; most studies utilize the minimal diameter of 3 mm to exclude small perivascular spaces. Studies also use an upper size limit of 15 mm, to differentiate lacunar infarcts from larger subcortical infarcts that reflect the involvement of more than one penetrating vessel (or cortical branch occlusions). This is due to tissue loss in older infarcts, and inflammation in the acute stage in the newer infarcts (Wardlaw et al., 2013b). Furthermore, many studies use the term “subclinical brain infarcts” or “silent brain infarcts,” where lacunar infarcts are grouped together with cortical infarcts, ignoring their distinct etiologies. Typically, the associations with presence or absence of infarcts are studied, although the number and location are also considered.

In this review article, we consider studies that group together all brain infarcts and emphasize results related to lacunar infarcts when defined by the study. Though many studies group all brain infarcts together, most also report that the majority of these silent infarcts are lacunar in nature.

### Etiology and Pathological Correlates

Pathologically, lacunar infarcts manifest as a result of lypohyalinosis and/or microatheroma of penetrating arteries, usually due to systemic hypertension or diabetes, and thus have a similar etiology to WML (Fisher, 1982; Prabhakaran et al., 2008; Knopman et al., 2011; Wardlaw et al., 2013b). Other mechanisms of disease include small emboli, atherosclerosis, and loss of endothelial integrity (Fisher, 1982; Wardlaw et al., 2003, 2009; Romero et al., 2009). Measures of subclinical carotid atherosclerosis have been associated with a higher incidence of lacunes (Brisset et al., 2013) and greater presence of subclinical brain infarcts (not excluding cortical infarcts; Romero et al., 2009; Caughey et al., 2018). Also, wider carotid lumen diameter has been related to a higher incidence of lacunes (Brisset et al., 2013), which may represent a compensatory response of the vessel to wall stiffness due to excessive extracellular matrix turnover, a lack of vascular smooth muscle cell proliferation, or apoptosis.

### Epidemiology

The prevalence of lacunar infarcts varies between studies, but are generally associated with increasing age across all studies. The Cardiovascular Health Study (CHS) reported a 78.2% prevalence of infarct-like lesions in deep nuclear location, and a 10.1% prevalence in the posterior fossa (Bryan et al., 1997). The CHS has also reported incidence data on lacunar infarcts, finding that 59.8% of those with lacunes had a single lacune only, while 15.8% of those with infarcts had multiple lacunes only (Longstreth et al., 2002). The Northern Manhattan Study, consisting mostly of Hispanic/Latino participants, reported a prevalence of silent brain infarcts of 16%, and 82.9% were classified as subcortical (Prabhakaran et al., 2008; Wright et al., 2017). In the Rotterdam Study, the prevalence of silent brain infarcts (including cortical) was 7.2% (Vernooij et al., 2007), and of the 217 participants with silent brain infarcts, 202 had lacunar infarcts specifically (Vermeer et al., 2003). In aggregate, most subclinical brain infarcts are lacunar in nature.

### Cognition

Lacunar infarcts have been associated with reduced cognitive performance in population-based cohort studies of elderly people, but few studies examined non-demented samples. Most studies examine these associations in multivariable models adjusting for age, so associations observed are independent of aging. A Japanese-based study found that multiple lacunar infarcts were associated with frontal lobe dysfunction specifically (Koga et al., 2009), which is consistent with a US-based cohort study (Knopman et al., 2015). The Rotterdam Study found that silent thalamic infarcts were specifically associated with steeper declines in memory performance, while nonthalamic infarcts were related to steeper declines in psychomotor speed (Vermeer et al., 2003). However, these studies are limited in terms of racial and ethnic diversity, which decreases their generalizability to the U.S. Overall, lacunar infarcts appear to be an important contributor to vascular cognitive impairment in population-based elderly cohorts, but more work is warranted to examine cognitive performance in non-demented elderly, especially in racial/ethnic minorities who are at higher risk for developing dementia and vascular disease compared to non-Hispanic whites (Mayeda et al., 2016; Benjamin E. J. et al., 2018).

## Perivascular Spaces

### Definition and Measurement

Also called Virchow-Robin spaces, perivascular spaces are extensions of the extracerebral fluid that surround small arteries and arterioles as they perforate the brain surface (Groeschel et al., 2006; Wardlaw et al., 2013b). They are not seen on conventional neuroimaging when small. However, the greater the resolution MRI is acquired, the more evident and more numerous they appear to be. Larger spaces located at the base of the brain become increasingly apparent with aging (Groeschel et al., 2006; Wardlaw et al., 2013b). The STRIVE panel defines dilated perivascular spaces as fluid-filled spaces that follow the typical course of a vessel as it travels through the gray or WM (Wardlaw et al., 2013b). They can be confused for lacunar infarcts, and therefore, their diameter should be less than 3 mm when imaged perpendicular to the course of the vessel (Wardlaw et al., 2013b). Perivascular spaces tend to be more prominent in the inferior basal ganglia area, where they can reach up to 20 mm, even causing some mass effect (Wardlaw et al., 2013b). A recognized lesion known as the infraputaminal lacune is an enlarged perivascular space in the subinsular region and can be mistaken for lacunar infarction, but pathological data have shown it to be of non-vascular origin (Pullicino et al., 1995). Measurement of perivascular spaces also heavily depends on MRI resolution, and therefore, estimates may widely vary depending on the strength of MRI used (De Guio et al., 2016). The relevance of perivascular spaces as an MRI marker of cognitive aging remains understudied. Researchers recently attempted to establish uniform criteria for identifying perivascular spaces through population-based studies (Adams et al., 2015). As with other CVSD markers, the presence, as well as the location of these spaces, are important classifiers for research, and it is hoped this biomarker will be utilized in clinical practice. Data on perivascular spaces in large population-based studies is extremely limited, especially in non-demented samples.

### Etiology and Pathological Correlates

Dilated perivascular spaces are thought to be caused by increased fluid exudation due to greater vascular permeability, obstruction of the lymphatic drainage system, or parenchymal atrophy (Groeschel et al., 2006; Adams et al., 2015; Ramirez et al., 2016). They have been pathologically correlated with pro-oxidative enzyme activation and complement activation in CAA (van Veluw et al., 2016), a vascular entity highly related to Alzheimer’s dementia.

The cause of dilated perivascular spaces is still under debate. They have been associated with other vascular risk factors for CSVD, such as elevated blood pressure (Klarenbeek et al., 2013; Yakushiji et al., 2014; Yao et al., 2014; Gutierrez et al., 2015) and large-vessel abnormalities (Gutierrez et al., 2013; Riba-Llena et al., 2018), however these results are not consistent in the literature (Bouvy et al., 2016). Thus although perivascular spaces seem to share similar risk factors as other measures of CSVD, more research is warranted.

### Epidemiology

Few population-based, epidemiological cohort studies have explored the prevalence or incidence of perivascular spaces, and moreover, these studies differ in terms of detection methods and data reported. However, across all studies, the load of perivascular spaces is significantly associated with greater age. The 3C-Dijon study reported that 28.5% of their non-demented and stroke-free cohort had 1–2 hippocampal dilated perivascular spaces, while 16% had >2 hippocampal dilated perivascular spaces (Yao et al., 2014). The Northern Manhattan Study, a mostly Hispanic/Latino elderly cohort, reported that 48% of their stroke-free sample had a perivascular spaces score of >4, which represents greater brain involvement (Gutierrez et al., 2013). A Japanese study of a neurologically healthy cohort reported prevalence rates for dilated perivascular spaces located in the basal ganglia and the centrum semiovale. It found that in the basal ganglia about 80% of their participants had mild, 11% had moderate, and about 3% had frequent or severe perivascular spaces. In the centrum semiovale, about 26% had mild, 51% had moderate, and 23% had frequent or severe perivascular spaces (Yakushiji et al., 2014). Overall, the reported prevalence varies tremendously in the literature, which is due in part to the lack of large, epidemiologic cohorts measuring this vascular entity in diverse populations.

### Cognition

The association between perivascular spaces and cognitive performance in the elderly has yet to be completely explored, and data in non-demented samples is especially limited. The results from studies of both population- and clinic-based samples are mixed. In the Age, Gene/Environment, Susceptibility-Reykjavik Study, dilated perivascular spaces were related to faster cognitive decline, particularly in processing speed (Ding et al., 2017a). In the community-based 3C-Dijon MRI Study of dementia- and stroke-free participants, dilated perivascular spaces were not related to cognitive performance (Yao et al., 2014). These findings are consistent with some smaller studies of clinical populations with cognitively mixed participants (Benjamin P. et al., 2018), but inconsistent with others (Huijts et al., 2014). Though not an epidemiologic sample, a small study of healthy older men found that dilated perivascular spaces were associated with worse cognitive function (Maclullich et al., 2004). Further research in larger cohorts is needed to understand the etiology of perivascular spaces and if they contribute uniquely to vascular cognitive impairment.

## Cerebral Microbleeds

### Definition and Measurement

MRI techniques that detect magnetic susceptibility allowed the recognition of CMBs, and this has improved as the technology has advanced (Greenberg et al., 2009). CMBs are defined as small round foci of signal void on T2*-weighted imaging measuring generally 2–5 mm in diameter, but sometimes up to 10 mm (Wardlaw et al., 2013b). Differences in the bulk magnetic susceptibility of tissue give rise to inhomogeneities in the magnetic field produced by the MR system. This results in an apparent decay of transverse magnetization (T2*) at a rate faster than that of spin-spin interactions (T2). Oxyhemoglobin is slightly diamagnetic and very similar to the neuropil, whereas deoxyhemoglobin, methemoglobin, and hemosiderin are paramagnetic owing to unpaired electrons. Red blood cells containing hemoglobin extravasate and undergo degradation in the perivascular space and parenchyma. The hemoglobin shifts the magnetic field over distances much greater than the underlying microscopic pathology, and in a sense, amplifies the effect to allow visualization in an MR image sensitized to T2*. Sequences such as gradient recalled echo (GRE), susceptibility weighted imaging (SWI), or practically any echo-planar weighted image such as diffusion weighted imaging (DWI) or dynamic susceptibility contrast (DSC) perfusion weighted imaging suffer from signal drop out in regions containing the blood degradation products.

Although initially associated with the presence of a concomitant lobar hemorrhage, they have also been detected in community-based samples. They are related to bleeding-prone microangiopathy of different origins, with the imaging findings corresponding to hemosiderin-laden macrophages (Fazekas et al., 1999; Shoamanesh et al., 2011). Hemosiderin is a blood breakdown product that causes magnetic susceptibility-induced dephasing, or loss of orientation of the nuclei due to relaxation of the signal, leading to T2* signal loss (Viswanathan and Greenberg, 2011).

The location of CMBs differ by etiology, with a cortical (or lobar) location suggesting amyloid angiopathy, and deep locations (i.e., in the basal ganglia or thalamus) suggesting hypertensive vasculopathy (Knudsen et al., 2001; Greenberg et al., 2009). CAA is diagnosed and subtyped using the Boston criteria, which considers clinical and pathological data for diagnosis and includes the identification of microbleeds (Knudsen et al., 2001). Additionally, many of these studies also examine the number of CMBs present, though estimates for these analyses become less precise as the number of participants with >1 CMB is usually low.

**Figure 1:**
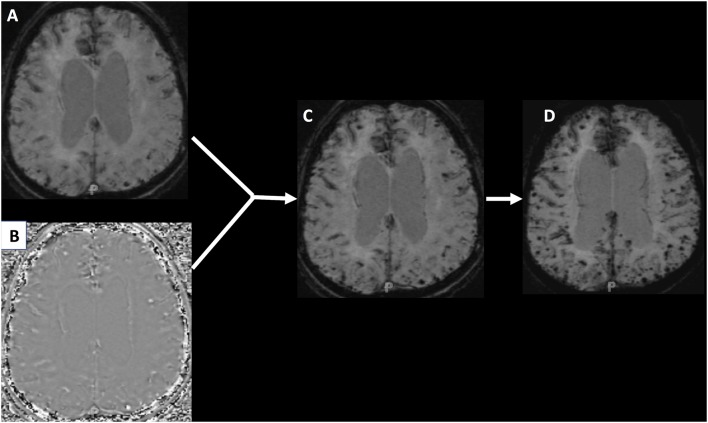
Panel **(A)** shows a traditional gradient recalled echo (GRE) image of a patient with hemosiderin deposition. Panel **(B)** is a phase image that when manipulated and integrated with the GRE produces the susceptibility weighted image (SWI) seen in panel **(C)**. Panel **(D)** is a minimum intensity projection (MIP) image that makes the cerebral microbleeds (CMBs) even more conspicuous.

Differences in MRI techniques and strength continue to be an issue in detecting CMB prevalence and incidence. The recent introduction of SWI into clinical practice has further improved the quantification of CMBs, but make it difficult to compare studies that use different techniques. SWI uses additional spin phase data from the MRI acquisition to enhance the appearance of CMBs and remove venous artifacts (Haacke et al., 2009). This can lead to an increase in the burden of CMBs detected when compared with traditional GRE imaging. Figure 1 shows how when a GRE image is adapted into an SWI, hemosiderin deposition becomes more evident and the number of CMBs appear to increase. Further, definitions of CMBs have varied over time and standardization of the imaging definition will be important for future studies.

### Etiology and Pathological Correlates

Many processes that weaken cerebrovascular integrity, and therefore increase the risk of bleeding, have been associated with CMBs. Common with other CSVD entities, elevated blood pressure is associated with CMBs, especially in deep locations, since deep CMBs are usually caused by hypertensive vasculopathy (Roob et al., 1999; Vernooij et al., 2008; Poels et al., 2010, 2011; Romero et al., 2014; Wiegman et al., 2014; Akoudad et al., 2015; Del Brutto et al., 2015). Further, systemic vasculopathies related to hypertension, including carotid stenosis, carotid intima-media thickness, and arterial stiffness, have also been associated with CMB presence (Poels et al., 2012b; Romero et al., 2016).

The other important etiology of CMBs is CAA, especially when CMBs are exclusively lobar or cortical. The APOE ε4 allele, a factor strongly related to amyloid angiopathy (Caselli et al., 2010; Tai et al., 2016), has been related to the increased prevalence and incidence of lobar location of CMB in several studies (Vernooij et al., 2008; Poels et al., 2010, 2011; Romero et al., 2014; Graff-Radford et al., 2017). Similar to CAA hemorrhages, lobar CMB tends to occur more frequently in the temporal lobe (Mesker et al., 2011). Additionally, APOE alleles ε4 and ε2 are more strongly related to clustering of lobar CMB when compared to carriers of the ε3 allele (Loehrer et al., 2014). Despite evidence for two separate etiologies, mixed pathology has also been described in several of these studies, which is not surprising given the older age of these participants. CMBs do not yet have diagnostic value with regard to CAA or hypertensive vasculopathy (Martinez-Ramirez et al., 2015).

Finally, systemic inflammation has also been suggested as a mechanism for CMB due to the microglial mediated clearance of blood products post-bleed that results in a detectable hypointense lesion on MRI. Romero et al. (2012) found that in subjects with at least 1 APOE ε2 or ε4 allele, high LpPLA2 levels, a known marker of vascular inflammation, was associated with deep CMB. In a subsequent report of the same cohort, CMB presence was associated with elevated levels of circulating tumor necrosis factor receptor 2 (TNFR2) and myeloperoxidase (Shoamanesh et al., 2015).

### Epidemiology

Several epidemiologic studies have estimated the prevalence of CMBs in the general population, as well as its relation to age and vascular risk factors. Generally, greater age is related to greater odds of having a CMB. Prevalence rates differ widely across reports and range between 4.7% and 24.4% (Roob et al., 1999; Jeerakathil et al., 2004; Vernooij et al., 2008; Poels et al., 2010, 2011; Wiegman et al., 2014; Akoudad et al., 2015; Del Brutto et al., 2015; Caunca et al., 2016; Ding et al., 2017b; Graff-Radford et al., 2017; van Leijsen et al., 2017) due to the heterogeneity in the ages of participants, MRI techniques, and CMB definitions. For example, the Framingham Study initially found a prevalence of CMB of 4.7% (Jeerakathil et al., 2004). In a subsequent report, the prevalence for CMB was 8.8%, almost double their previous report, despite the mean age being similar (Romero et al., 2014). The difference in prevalence may be explained by a difference in MR strength (1.0T vs. 1.5T) since increased magnetic field strength enhances CMB detection (Scheid et al., 2007; Stehling et al., 2008). In a more diverse sample, the Northern Manhattan Study found a prevalence rate of 5% for CMBs (Caunca et al., 2016). The Washington Heights/Inwood Columbia Aging Project, also based in Northern Manhattan, found a prevalence of 27%; however, their study used stratified sampling based on Medicare enrollees (aged 65 and older), while the Northern Manhattan Study randomly sampled a younger population (aged 50 and older; Wiegman et al., 2014). Further, the former study had a smaller sample size than the latter, and CMB detection methods differed as well. In contrast, the Rotterdam Study has reported a prevalence of CMBs ranging from 15.3% to 28% across several years (Vernooij et al., 2008; Poels et al., 2010, 2011). Similarly, the Atherosclerosis Risk in Communities Study found an overall prevalence of 24% in their biracial cohort using a 3T-strength MR machine (Graff-Radford et al., 2017). Generally, CMBs are more commonly located in lobar vs. deep locations (Jeerakathil et al., 2004; Caunca et al., 2016; Ding et al., 2017b; Graff-Radford et al., 2017), especially in the temporal lobe (Mesker et al., 2011). The incidence of CMBs also varies, but prospective studies in community-based populations are limited. The Rotterdam Study found a 3-year incidence rate of 10% for new CMBs (Poels et al., 2011), while the RUN DMC cohort exhibited an annual 2.2% incidence rate for CMBs over a 9-year period (van Leijsen et al., 2017). In an Ecuadorian, rural sample, the prevalence of CMBs was 11% (Del Brutto et al., 2015). In the Age, Gene/Environment Susceptibility-Reykjavik Study, the prevalence of CMBs was 16.8% (Ding et al., 2017b). Chinese population-based studies have found 10.1% (Han et al., 2018) and 32.3% (Hilal et al., 2014) of CMBs, respectively.

### Cognition

The presence of one or more CMBs has been associated with decreased global cognitive performance, independent of age, but there is relatively limited evidence in non-demented, epidemiologic samples (Poels et al., 2012a; Ding et al., 2017b). Domain-specific cognitive performance has also been related to CMBs in non-demented samples (Poels et al., 2012a; Meier et al., 2014; Akoudad et al., 2016; Paradise et al., 2019), but results are mixed in terms of which CMB location is related to which cognitive domain. Overall, CMBs, especially lobar CMBs, appear to affect mostly executive function processes, which is consistent with the clinical presentation of vascular cognitive impairment.

## Blood-Brain Barrier Disruption

### Definition and Measurement

The BBB is a dynamic interface between the cerebral circulation and the central nervous system that limits and regulates the movement of cells and molecules between these two spaces *via* an interdependent network of cells and cell structures (Sandoval and Witt, 2008). An intact BBB generally acts to protect the brain; however, facilitated permeability of the BBB also plays an important role in healthy brain physiology, such as during sleep that controls the opening of the BBB (Pan and Kastin, 2017).

Gadolinium-enhanced MRI is the modality most commonly used to assess BBB integrity. Gadolinium based contrast agents (GBCAs) administered intravenously do not normally cross an intact BBB. When present, GBCAs dramatically shorten the T1 and T2 relaxation time-constants providing a mechanism to “enhance” the signal in regions where the BBB is compromised. Clearance of GBCAs is through the kidneys and most agents have a half-life in the vasculature of a couple of hours.

**Figure 2:**
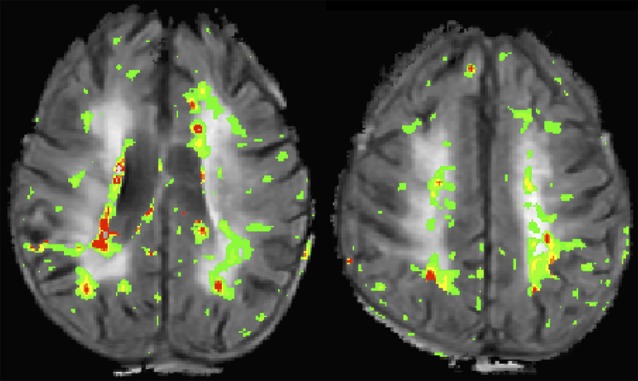
This image shows two slices from a flair magnetic resonance imaging (MRI) of a patient with confluent white matter hyperintensities (WMH). A blood-brain barrier (BBB) permeability heatmap derived from a dynamic susceptibility contrast (DSC) sequence has been superimposed in color. Increasing BBB permeability follows the color sequence green, yellow, orange, red.

The typical clinical assessment of the BBB involves T1-weighted imaging before and after administration of GBCAs. This approach is able to detect overt disruption of the BBB such as with brain tumors or multiple sclerosis, however, it is insensitive to more subtle gadolinium leakage. The most commonly used research tool for measuring BBB permeability is dynamic contrast enhanced (DCE) MRI which uses serial T1-weighted imaging to measure the transfer constant (Ktrans) during steady state. A more pragmatic approach uses DSC imaging, which is a modality commonly acquired for perfusion imaging in the clinical setting. Both DCE and DSC have been used to measure BBB disruption associated with CSVD (Taheri et al., 2011b; Arba et al., 2017). Figure 2 shows an example of a BBB permeability heatmap derived from a DSC sequence superimposed on a FLAIR sequence for a patient with confluent WMH.

### Etiology and Pathological Correlates

Dysfunction or disruption of the BBB is known to be associated with many diseases of the central nervous system (Weiss et al., 2009). BBB permeability also increases with normal aging but is accelerated in patients with WMH (Farrall and Wardlaw, 2009). White matter BBB disruption has been identified in patients suffering from lacunar stroke (Wardlaw et al., 2009), and vascular cognitive impairment (Taheri et al., 2011a). In patients with a history of lacunar stroke, BBB disruption is known to occur not only within the WMH but also in the normal appearing white matter (NAWM; Topakian et al., 2010). The pattern of WMH-associated BBB disruption tends to be located around the edges of WMH, stretching into the NAWM (Rosenberg et al., 2014; Huisa et al., 2015). It has been hypothesized, but not yet proven, that BBB disruption in the NAWM precedes the development of WMH (Wardlaw et al., 2013a).

BBB disruption has been suggested as an early stage in the cascade of events that leads to the development of WML (Rosenberg et al., 2016). Areas of the brain susceptible to intermittent hypoxia with reduced cerebrovascular autoregulation experience activation of the MMPs resulting in degradation of the endothelial basil lamina and tight junction proteins, opening the BBB. This hypoxic pro-inflammatory environment leads to the accumulation of free radicals and proteases resulting in myelin degradation through a non-immune mediated inflammatory demyelination (Rosenberg et al., 2016).

### Epidemiology

Most studies looking at the relationship between BBB disruption and CSVD have been performed in patients who were identified due to the occurrence of an acute ischemic event. An analysis of an imaging data registry of stroke patients found that an increasing burden of traditional biomarkers for CSVD (WML, CMB, etc.) was associated with an increase in measures of BBB disruption both in the region of the ischemic event as well as in areas remote from the infarction (Arba et al., 2017). Another analysis of stroke patients with WML found a dissociation between the traditional CSVD risk factor of hypertension and the occurrence of BBB disruption (Gupta et al., 2018).

The incidence and prevalence of BBB disruption in the general population of patients with subclinical CSVD have not been examined in large population-based studies to date. BBB disruption detected at the time of a stroke can persist for months after the initial event, suggesting that it is a chronic process rather than merely a manifestation of the acute event. In a small study of patients with known vascular dementia, BBB disruption was present on serial imaging and appeared to migrate over time (Huisa et al., 2015). Recent data from clinical samples also seems to suggest that MRI measures of BBB disruption are not related to age (Nation et al., 2019), but further studies in larger, population-based samples are needed to explore age-related associations.

### Cognition

Much of the research looking at BBB disruption has been focused on its relationship to the development of WML. It is possible that BBB disruption precedes the conversion of NAWM to WML. Since progressive WMLs are associated with cognitive decline, it is hypothesized that BBB disruption may be the earliest biomarker for identifying at-risk patients. This hypothesis is supported by one study that found BBB disruption preceded the development of post-stroke cognitive decline (Wardlaw et al., 2017). More recently, a smaller clinically-based study was conducted showing that BBB disruption was related to cognitive dysfunction, independent of Alzheimer’s Disease imaging biomarkers as well as after adjustment for age (Nation et al., 2019). Participants in this study were selected from Alzheimer’s Disease Research Centers, and included participants with early cognitive dysfunction. Thus, more work needs to be done in larger, representative samples to improve both internal and external validity.

## Diffusion Tensor Imaging

### Definition and Measurement

Diffusion tensor imaging (DTI) allows for 3-dimensional measurement of the displacement of water molecules within tissues as a result of Brownian motion caused by heat (Basser et al., 1994; Basser and Pierpaoli, 1996; Alexander et al., 2007). Water diffusing in tissue interacts with cell membranes and other microstructural features that infer with, and decrease, the net displacement in comparison to that of bulk fluid. Some of these features, such fiber tracts formed by axons, have coherent structural orientation which in turn is reflected in the net displacement measured by DTI. The magnitude and the directional dependency can be decomposed and used from the DTI data to infer the structural integrity of the tissue.

Commonly, two measures of WM microstructural integrity are used: the MD and FA. The MD reflects the overall diffusion within a voxel or region of interest, with greater MD indicating more microstructural integrity loss (Soares et al., 2013). On the other hand, the FA reflects the degree of anisotropy (ranges from 0 to 1) or restriction of the water diffusion in a single direction. Values of FA approaching 1 indicate that the water diffusion is occurring in a single direction, which indicates better microstructural integrity (Soares et al., 2013).

### Etiology and Pathological Correlates

Mechanisms of microstructural WM integrity loss are still being elucidated, and the etiology of WM microstructural integrity loss is multifactorial. Generally, vascular risk factors are strongly related. In a middle-aged, biracial sample, hypertension and greater time spent in sedentary activities were related to lower FA (Launer et al., 2015). Similarly, greater systolic blood pressure was related to decreased FA and increased MD, especially in the anterior corpus callosum and inferior fronto-occipital fasciculus in a mostly White sample (Maillard et al., 2012). More recently, studies have shown that both midlife and late-life vascular risk factors were related to worse microstructural integrity among older adults (Wang et al., 2015; Power et al., 2017). Relatedly, behavioral risk factors, such as smoking and diet, are also related to WM microstructural integrity (Gons et al., 2011; Launer et al., 2015; Gu et al., 2016). Finally, serum measures of systemic inflammation have also been associated with lower FA and greater MD, suggesting a potential inflammatory mechanism for microstructural integrity loss (Walker et al., 2017, 2018).

Studies have shown that non-demented APOE ε4 allele carriers exhibit increased MD, suggesting that the APOE ε4 allele may affect cerebral lipid metabolism and subsequently the integrity of the myelin of these WM tracts (Kljajevic et al., 2014; Operto et al., 2018). In a smaller study of cognitively normal participants from AD Research Centers, a greater phosphorylated tau-Aβ42 ratio was related to higher MD (Racine et al., 2019). These data are also consistent with the idea that microstructural integrity loss might be an earlier marker of pathological brain aging than other markers of CSVD.

Microstructural WM integrity has been studied in the context of WM lesions as seen on FLAIR images. Generally, in cognitively normal samples, greater WMHV is related to lower FA (Seiler et al., 2018). Importantly, WM microstructural integrity loss has been observed outside WMHs in aging populations, suggesting that WMHs represent the most severe damage to the WM, but WM injury continues outside these lesions (Maillard et al., 2011, 2014).

### Epidemiology

In a sample of participants with ages ranging across the life-course, non-linear associations observed between WM microstructural integrity and age were observed, highlighting the need to consider changes in WM integrity in the context of different age spans (Slater et al., 2019). Similarly, in a sample of aging adults (age range 46–100 years), non-linear associations of FA and MD were observed with increasing age (Vinke et al., 2018). Prospective cohort studies have observed decreasing FA and increasing MD over 2-year follow-up period among older adults with an average age of about 70 years (de Groot et al., 2016). Among non-demented oldest-old (i.e., aged 90 years or older), age-related changes in WM microstructural integrity (as measured by MD and FA) has been observed in regions important to dementia risk (Bennett et al., 2017). In younger samples, greater age was also related to lower FA and greater MD (Maillard et al., 2012; Launer et al., 2015).

### Cognition

Generally, studies have observed that greater MD and lower FA were related to faster decline in global cognition among non-demented older adults (Tuladhar et al., 2015; Wang et al., 2015), especially among APOE ε4 allele carriers (Wang et al., 2015). Domain-specific associations have also been observed in a prospective cohort studies of non-demented and stroke-free older adults, where both global and tract-specific FA and greater MD was related to worse processing speed, executive function, and motor speed (Vernooij et al., 2009; Cremers et al., 2016; Seiler et al., 2018). Others have found similar associations and suggest that the effect of upstream factors, such as diet, on cognitive decline may be mediated by WM microstructural integrity (Gu et al., 2016). However, more work needs to be done, especially in epidemiologic studies.

## Conclusion

In summary, most CSVD markers, especially WM lesion load, subclinical infarcts, and diffusion measures, are related to cognitive decline independent of age. More research needs to be done to examine age-related cognitive changes in relation to BBB disruption and changes in diffusion-based markers of microstructural integrity. Understanding the relationship between normal aging and cognitive decline is a challenging endeavor. However, MRI markers of CSVD may offer a critical clue to understanding this relationship. Large, epidemiological neuroimaging studies have the potential to provide valuable information to guide research. The use of population-based cohorts ensures generalizability and affords the opportunity to study a variety of risk factors and outcomes related to neuroimaging markers. Despite the significant work that has been done with established MRI markers, further epidemiological research is warranted in understudied markers, such as perivascular spaces and BBB disruption. While work in aging populations can inform researchers and clinicians of the prevalence and incidence of these markers in age-related cognitive changes, future studies should focus carefully on excluding cognitively abnormal participants particularly in studies that use cognitive performance as an outcome. This will facilitate the study of how these MRI markers relate to cognitive impairment in the context of normal aging.

## Author Contributions

CW contributed to the conception of this review article. MC and AL-B conducted the literature review and wrote the first draft of the manuscript. LL and RL wrote sections of the manuscript. All authors contributed to manuscript revision and read and approved the submitted version.

## Conflict of Interest Statement

CW receives royalties for 2 chapters on Vascular Dementia from UpToDate.
